# 

**Published:** 1883-11

**Authors:** 


Vol. IV. NOVEMBER, 1883. No. 11.

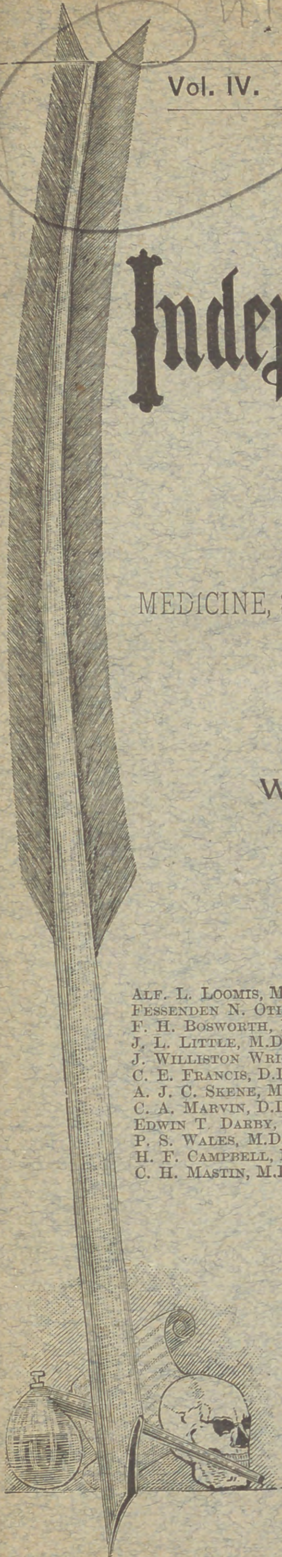

### THE Independent Practitioner:

A MONTHLY JOURNAL,

LIBRARY
INDEXED
S.G.O.

DEVOTED TO

MEDICINE, SURGERY, OBSTETRICS, DENTISTRY, PATHOLOGY AND POPULAR SCIENCE.

W. C. BARRETT, M. D., D. D. S.,

EDITOR.

COLUMBIA PUBLISHING COMPANY.

$2.50 Per Annum in Advance. - - Single Copies, 25 Cents.

Entered at the Post-Office, Buffalo, N. Y., as Second-Class matter.

